# Return of warm conditions in the southeastern Bering Sea: Phytoplankton - Fish

**DOI:** 10.1371/journal.pone.0178955

**Published:** 2017-06-28

**Authors:** Janet T. Duffy-Anderson, Phyllis J. Stabeno, Elizabeth C. Siddon, Alex G. Andrews, Daniel W. Cooper, Lisa B. Eisner, Edward V. Farley, Colleen E. Harpold, Ron A. Heintz, David G. Kimmel, Fletcher F. Sewall, Adam H. Spear, Ellen C. Yasumishii

**Affiliations:** 1 Alaska Fisheries Science Center, NOAA, National Marine Fisheries Service, Seattle, Washington, United States of America; 2 Pacific Marine Environmental Laboratory, Seattle, WA, United States of America; 3 Alaska Fisheries Science Center, Auke Bay Laboratories, NOAA, National Marine Fisheries Service, Juneau, Alaska, United States of America; Helmholtz-Zentrum fur Ozeanforschung Kiel, GERMANY

## Abstract

In 2014, the Bering Sea shifted back to warmer ocean temperatures (+2 ^o^C above average), bringing concern for the potential for a new warm stanza and broad biological and ecological cascading effects. In 2015 and 2016 dedicated surveys were executed to study the progression of ocean heating and ecosystem response. We describe ecosystem response to multiple, consecutive years of ocean warming and offer perspective on the broader impacts. Ecosystem changes observed include reduced spring phytoplankton biomass over the southeast Bering Sea shelf relative to the north, lower abundances of large-bodied crustacean zooplankton taxa, and degraded feeding and body condition of age-0 walleye pollock. This suggests poor ecosystem conditions for young pollock production and the risk of significant decline in the number of pollock available to the pollock fishery in 2–3 years. However, we also noted that high quality prey, large copepods and euphausiids, and lower temperatures in the north may have provided a refuge from poor conditions over the southern shelf, potentially buffering the impact of a sequential-year warm stanza on the Bering Sea pollock population. We offer the hypothesis that juvenile (age-0, age-1) pollock may buffer deleterious warm stanza effects by either utilizing high productivity waters associated with the strong, northerly Cold Pool, as a refuge from the warm, low production areas of the southern shelf, or by exploiting alternative prey over the southern shelf. We show that in 2015, the ocean waters influenced by spring sea ice (the Cold Pool) supported robust phytoplankton biomass (spring) comprised of centric diatom chains, a crustacean copepod community comprised of large-bodied taxa (spring, summer), and a large aggregation of midwater fishes, potentially young pollock. In this manner, the Cold Pool may have acted as a trophic refuge in that year. The few age-0 pollock occurring over the southeast shelf consumed high numbers of euphausiids which may have provided a high quality alternate prey. In 2016 a retracted Cold Pool precluded significant refuging in the north, though pollock foraging on available euphausiids over the southern shelf may have mitigated the effect of warm waters and reduced large availability of large copepods. This work presents the hypothesis that, in the short term, juvenile pollock can mitigate the drastic impacts of sustained warming. This short-term buffering, combined with recent observations (2017) of renewed sea ice presence over southeast Bering Sea shelf and a potential return to average or at least cooler ecosystem conditions, suggests that recent warm year stanza (2014–2016) effects to the pollock population and fishery may be mitigated.

## Introduction

Sea-ice presence in the spring establishes the annual oceanographic conditions over the Bering Sea shelf. Variations in spring sea-ice characteristics (thickness, extent, timing of advance/retreat) lead to physical ocean conditions that have been previously described as thermally average, warm, or cold over the shelf during summer and autumn. Historically (prior to 2000), these physical fluctuations have been short-term (1–2 years) and there were few effects of punctuated variability to overall ecosystem structure and functioning. However, in the two last decades the duration of thermal stability has become significantly protracted (4–6 years) and the Bering Sea has shifted from experiencing short-term, punctuated variability in thermal ocean conditions to undergoing longer-term stanzas of sustained warm or cold periods. This change was first observed in the early 2000s when a protracted warm anomaly heated the southeast Bering Sea shelf (SEBS; denoted as the shelf south of 60 ^o^N) ~2 ^o^C over a period of 5 years (2001–2005; Warm Years). During this time sea ice extent was retracted far northward, allowing the SEBS shelf a period of gradual, sustained heating. The full impact of the Warm Year stanza to other components of the ecosystem was realized only later, when the age 3+ biomass of the commercially valuable walleye pollock (*Gadus chalcogrammus*; hereafter, pollock) plummeted over 40% between 2004 and 2008 [1]. The large decline was unexpected, but data from integrated ecosystem surveys connected the decline to climate mediated-impacts on fitness and survival of pollock early life stages [2]. Specifically, it was determined that while single warm years had little or even weakly positive effects on pelagic production, multiple warm years led to a restructuring of the zooplankton community to smaller (< 2 mm prosome length), lipid-poor [2] taxa (i.e., *Oithona* spp. and *Pseudocalanus* spp. copepods) which provided an inadequate food supply to age-0 (first year juvenile) pollock provisioning for winter [3]. Ultimately the consequence of the Warm Year stanza was a rapid and dramatic decline in pollock recruitment. Sea ice returned to the southeast Bering Sea shelf in 2006, ushering in a new stanza of cold sea temperatures (2007–2013; Cold Years). A shift in zooplankton community prey base back to large-bodied (> 2 mm prosome length), lipid-rich species (i.e. *Calanus* spp. copepods and euphausiids) occurred [4], and age-0 pollock feeding on these prey experienced a significant improvement in energetic condition and higher over-winter survival [5]. There was a complete recovery of the age-3+ pollock biomass by 2013 [1].

Integrated ecosystem survey data collected during 2014 identified an emerging shift in ocean conditions from the recent prolonged cold period back to a warm Bering Sea. Sea ice thickness and ice extent were reduced in winter/spring 2014 and summer bottom water temperatures were the warmest (4–5 ^o^C) observed since 2005. A large coccolithophore bloom was observed [6], a phenomenon associated with stratified ocean conditions and nutrient-poor water. In addition, age-0 pollock vertical distribution shifted to near surface waters [7] as was seen during the last warm period. These observations suggested that the southern Bering Sea ecosystem was changing, but whether the variability would be a short-term, Warm Year anomaly with limited trophic implications, or the first in a series of consecutive Warm Years (stanza) that could precipitate a multi-trophic ecosystem shift, remained unresolved. Recognizing the importance of capturing ecosystem change in progress, the National Oceanic and Atmospheric Administration’s National Marine Fisheries Service initiated field research in 2015 and 2016 to fully characterize the Bering Sea ecosystem’s response to a thermal warming event in progress. The aim was to document either ecosystem resiliency, if warming was determined to be punctuated, short-term, and non-sequential, or ecosystem vulnerability, if warming was determined to be sequential, persistent, and sustained. Here we report findings from these research surveys and we evaluate whether observed physical oceanographic characteristics in the Bering Sea 2014–2016 have meaningful implications for pollock recruitment dynamics and ecosystem structure and functioning.

## Materials and methods

### Study areas, sampling and ocean conditions

**Fig 1:**
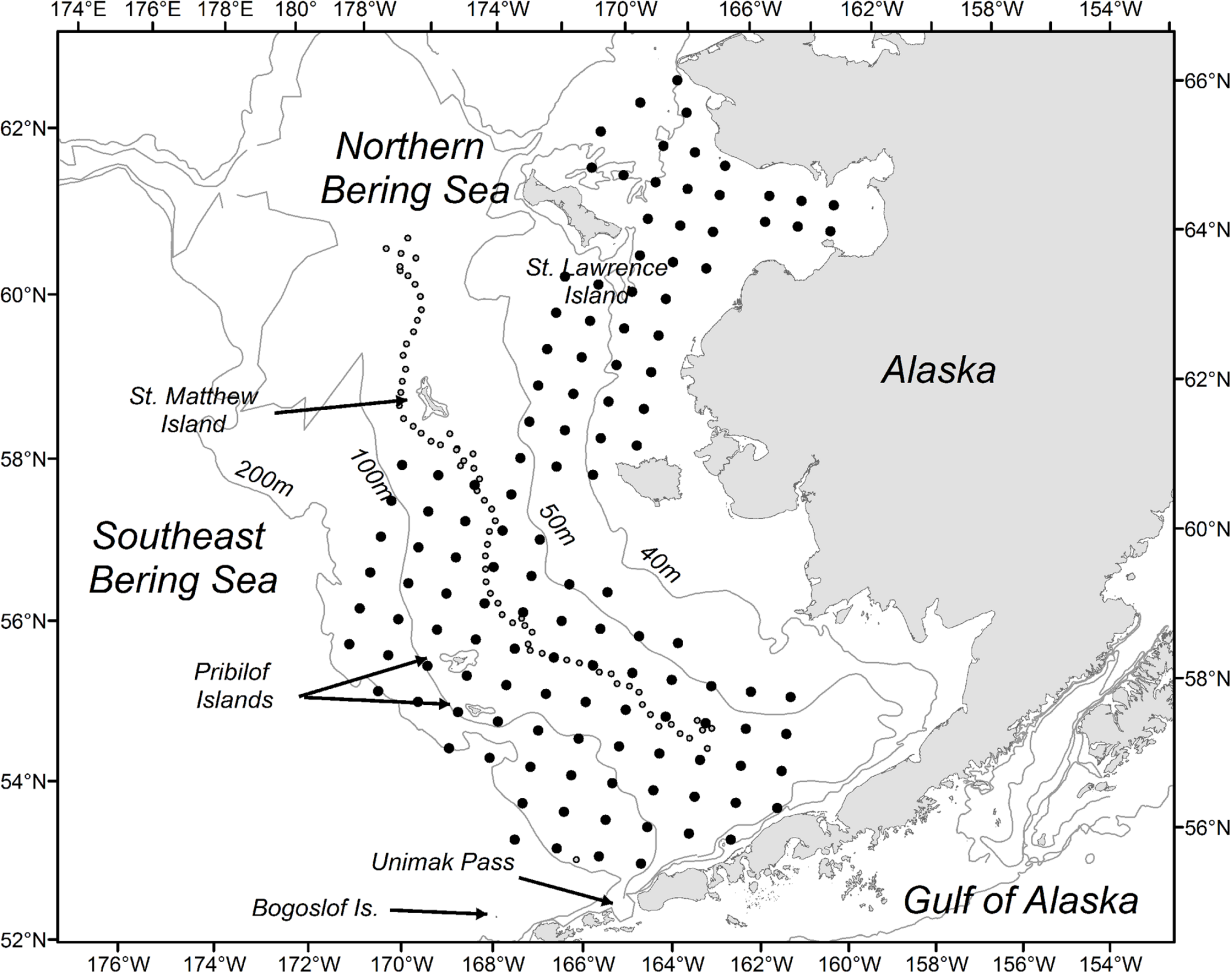
Survey area and geographic extent. Schematic diagram of the southeast Bering Sea and Northern Bering Sea with seasonal sampling in 2015 and 2016 overlaid. Dark circles indicate gridded survey areas; light, small circles indicate latitudinal sampling along the 70 m isobath.

Field Collection Permits were authorized by the National Marine Fisheries Service/Alaska Fisheries Science Center Regional Office and by the Alaska Department of Fish and Game. A series of multi-disciplinary oceanographic research cruises to the SEBS and Northern Bering Sea (NBS) were conducted April-October 2015 and 2016 (Fig 1, Table 1). Sampling along selected transects or over gridded stations included physical measurements (S1 Table) and biological sampling inclusive of at-sea chlorophyll a (Chl*a*) and phytoplankton taxa measurements (2015 only, S2 Table), at-sea mesozooplankton analyses (S3 Table), surface and midwater trawling for age-0 pollock (S4 Table), acoustic backscatter (S5 Table) and diet analyses of collected pollock (S6 Table). In the laboratory, expedited processing of field-collected phytoplankton samples for Chl*a*, and analysis of age-0 pollock for energetic content (S7 Table), provided estimates of phytoplankton biomass and nutritional status of zooplanktivorous fish, respectively. These data offered rapid indices of the Bering Sea ecosystem during an oceanographic shift.

**Table 1:** Surveys and sampling.

Cruise	Date	Sampling Methodology
DY15-04	April 24 –May 10, 2015	CTD, moorings, satellite-tracked drifters, Chl*a*, zooplankton (RZA)
AE15-01	September 1 –September 12, 2015	CTD, surface trawl, age-0 pollock diet analyses
DY15-08	September 5 –September 18, 2015	CTD, Chl*a*, zooplankton (RZA), midwater trawl, age-0 pollock diet analyses
DY15-09	September 21 –October 6, 2015	CTD, moorings, Chl*a*, zooplankton (RZA), midwater trawl, age-0 pollock diet analyses, acoustics
DY16-06	May 3 –May 14, 2016	CTD, moorings, Chl*a*
DY16-07	May 17 –June 8, 2016	CTD, larval pollock, zooplankton (RZA)
AE16-01	August 26 –September 19, 2016	CTD, surface trawl, age-0 pollock diet analyses
DY16-09	August 22 –September 20, 2016	CTD, Chl*a*, zooplankton (RZA), midwater trawl, age-0 pollock diet analyses
DY16-10	September 24 –October 6, 2016	CTD, moorings, Chl*a*, zooplankton (RZA), acoustics

Oceanographic research surveys and associated at-sea sampling over the eastern Bering Sea shelf in 2015 and 2016.

Temperature data collected from mooring arrays, standard conductivity-temperature-depth (CTD) sensors (Sea-bird electronics (SBE) 911), and Fastcat (SBE 49) CTDs showed that bottom temperatures over the southeast Bering Sea shelf were among the warmest on record (2015: 4 ^o^C, 2016: 5 ^o^C). Despite significant warming in both years, there were differences in presence and extent of spring sea ice between 2015 and 2016. In 2015, spring conditions were marked by northwesterly wind events that advected sea ice southward as far as ~57.5 ^o^N. Sea ice advance may have been even further south but was prevented by the presence of warm water [8, 9, 10] that had been transported to the SEBS through Unimak Pass from the Gulf of Alaska. As such, while ocean conditions over the southern shelf were anomalously warm in 2015, a fairly large residual Cold Pool, a region of cold bottom water (<2.0 ^o^C) associated with sea ice presence [11] was present above ~57 ^o^N (Pribilof Islands, Fig 1) and persisted through the summer and autumn. Conditions in spring 2016 were markedly different. Weak north winds in 2016 precluded the advance of sea ice over the southern shelf, resulting in a sea ice edge that proceed no further south than ~59 ^o^N and resulted in a retracted Cold Pool (~59 ^o^N; St. Matthew Island, Fig 1) in summer and autumn. Despite these differences in Cold Pool size and extent, the SEBS shelf in both years was very warm, strongly stratified, and nutrient poor. A bloom of coccolithophores (*Emiliania huxleyi)*, a single-celled small (5 μm) phytoplankter with plates composed of calcium carbonate, was detected in September of both years, which gave the water a milky aqua-colored appearance and substantially reduced in–water light levels [6].

### Phytoplankton

Water samples were collected along the 70-m isobath from Niskin bottles mounted on a standard CTD rosette system during spring and late summer /early autumn (research cruises DY15-04, DY15-09, DY16-06, DY16-07, Table 1, Fig 1). Samples were collected from discrete water depths (0 to 50 m at 10 m intervals) and filtered through Whatman GF/F filters (nominal pore size 0.7 μm) to estimate total Chl*a*. Water samples were also filtered through polycarbonate filters (pore size 10 μm) to estimate large-fraction Chl*a* at a subset of stations. Filters were frozen (-80 ^o^C) at sea and analyzed in the laboratory within 6–9 months using standard methods [12]. Discrete depth Chl*a* concentrations (mg m^-3^) were integrated over the water column to estimate total integrated Chl*a* (mg m^-2^) at each station. In 2015, large phytoplankton taxa were also characterized from unpreserved (live) water samples using a benchtop imaging FlowCytobot (IFCB,[13]) at a subset of stations and depths. Briefly, the IFCB pulls the sample through an intake tube with 150 um mesh into a laminar flow field that allows single particles to flow past a Chl*a* florescence detector and camera. Particles are photographed if they fluoresce, and photos are electronically stored for later phytoplankton identifications. Most, but not all, water samples were concentrated (using a 10 μm mesh net) prior to IFCB analysis to obtain sufficient cell numbers. Phytoplankton taxa characterization was not conducted in 2016.

### Zooplankton

An on-board assessment of zooplankton community structure (Rapid Zooplankton Analysis, RZA) provided a preliminary, real-time index of zooplankton species composition and abundances along the 70 m isobath latitudinal gradient (Table 1, Fig 1). The method, which is a rough count of crustacean zooplankton taken from one side of a paired, 20 cm/60 cm bongo array (153 μm and 505 μm mesh, respectively), sorts plankton into coarse taxonomic categories using standard zooplankton sorting approaches. Categories for rapid identification include, but are not limited to, small copepods (< 2 mm total length; includes *Acartia* spp., *Pseudocalanus* spp. and *Oithona* spp.), large copepods (> 2 mm total length; includes *Calanus* spp. (*Calanus marshallae/glacialis*), *Metridia* spp., and *Neocalanus* spp.), and euphausiids (*Thysanoessa* spp.). These categories were chosen as they describe abundances of ecologically important species, they provide important prey sources for upper trophic zooplanktivores [5], and they are highly influenced by warm / cold oceanographic conditions [14, 15]. The euphausiid category included all life stages, though it should be noted that adult stages are not well sampled by bongo nets, therefore mainly juvenile stages were enumerated. Integrated abundances in each RZA category over the SEBS shelf were estimated using the volume filtered from each tow. Using this approach, the zooplankton community at selected latitudinal stations along the 70 m isobath on the southern Bering Sea shelf was assessed in 2015 and 2016 (DY15-04, DY15-09, DY16-07, DY16-10; Table 1). The RZA was not performed during the NBS surveys (NE15-01, NE16-01) due to logistical constraints.

### Fish

Age-0 pollock were collected from midwater trawls towed obliquely at gridded stations (30 nautical mile spacing) over the SEBS shelf from 162 to 171°W and from the Alaska Peninsula to 58°N (DY15-08, DY16-09; Table 1). The trawl mouth opening was 8–12 meters deep x 12–18 m wide with knotted nylon mesh decreasing from 6 inches at the trawl mouth to 1.5 inches in the codend with a 10 mm mesh codend liner. The trawl was fished from a footrope depth of 10 meters off bottom (or 200 meters maximum depth) to the surface with a warp retrieval rate of 10 m min^-1^. Collections were also made in the NBS from 58 ^o^N to 63 ^o^N during research cruises using a surface trawl which was originally designed for sampling juvenile salmon but has been used to estimate relative abundances of age-0 pollock [16, 17]. The surface trawl was a 198-m-long midwater rope trawl with hexagonal mesh wings, a 12-mm mesh liner in the codend, and a mouth opening of 55 m wide x 20 m deep. The trawl was towed horizontally through the water for 30 min at 6.5–9.3 km h^−1^, during daylight hours, and sampled the water column from 0–25 m depth. It should be noted that the NBS is comparatively shallow (30–60 m depth) so surface trawling did, in fact, sample much of the water column above the pycnocline (~30 m). On all surveys, age-0 pollock were enumerated and measured, diets were examined either on board or in the laboratory, and subsamples were frozen for later calorimetry.

Acoustic backscatter data were collected from DY15-09 and from DY16-10 using Simrad EK60 and analyzed using Myriax Echoview (v. 6.1). Standard sphere calibrations [18] conducted prior to the surveys to measure system performance did not reveal evidence of changes in sensitivity during the surveys. Five split-beam transducers (18, 38, 70, 120, 200), mounted along the vessel’s centerboard, were extended to 7.6 m below the surface. Acoustic data were collected at a rate of 2.5 ping s^-1^ and a pulse length of 0.512 ms. Results presented here were based from 38 kHz data (S_v_ integration threshold = -70 dB re m^-1^) which were analyzed from 12 m below the surface to 3 m of the sounder-detected bottom. Backscattering that was likely attributed to adult groundfishes were not included in the analysis. Since no trawling was conducted on these portions of the surveys echosign could not be verified; observations are reported in terms of backscatter (Nautical Area Scattering Coefficient, m^2^/nmi^2^).

Stomachs of either 10 (DY15-08, DY15-09, DY16-09) or 3 (AE15-01, AE16-01) randomly selected fish (<110 mm standard length, SL) were pooled and stomach contents were taxonomically identified and weighed by prey group [17]. Samples were collected from over most of the Bering Sea shelf, but logistical constraints precluded trawl sampling of some northern stations of the survey grid in both years. Where sufficient age-0 pollock were caught, an additional subsample of fish (*n* = 5) was frozen (-20°C) for biochemical and energetics analyses in the laboratory. From each haul, three (*n* = 3) fish representing the mean length at that station were weighed, dried, and combined for biochemical analyses (energy density, kJ/g dry mass) using bomb calorimetry. This procedure dries fish at 135 ^o^C, homogenizes them, and places them in a bomb calorimeter to measure the energy released from sample combustion. Full methodology is provided in [19]. Due to differences in towing protocols between the SEBS shelf and the NBS, energy content of age-0 pollock in the south reflect integrated water column estimates and those from the NBS reflect surface (0–25 m) estimates. As noted above however, surface trawls (0–25 m) in the NBS sampled a large portion of the water column due to the comparatively shallow water depth.

In order to compare results to time series data, abundances of age-0 pollock in surface waters were estimated with geospatial modeling methods using the Spatial DeltaGLMM package version 31 [20] in R statistical software [21]. Model specifications included using a lognormal distribution and estimated spatial variation for both encounter probability and positive catch rate net components, and a spatial resolution with 100 knots [20].

We were interested to learn whether age-0 pollock have the potential to move out of warm waters over the SEBS shelf to cooler northern waters associated with the Cold Pool. It is known that age-0 pollock adopt a metabolic energy-conserving behavior during times of limited food availability, moving to lower temperatures and reducing energy expense [22]. As such thermal cues over the SEBS shelf could prompt a migratory response. We evaluated the strength of thermal gradients using vector plots that visualized the magnitude and direction of bottom temperature gradients in the SEBS and NBS. Using MATLAB™ (R2016b), bottom temperature data were transformed into a gridded matrix using linear interpolation, followed by calculation of the Eastward (u) and Northward (v) directions of the vector field. A geospatial processing program (ESRI ArcGIS™) was used to create the temperature gradient vector plots by converting the u and v components to direction and magnitude. Temperatures were multiplied by (-1) to orient the vectors toward decreasing temperatures.

## Results

### Phytoplankton

**Fig 2:**
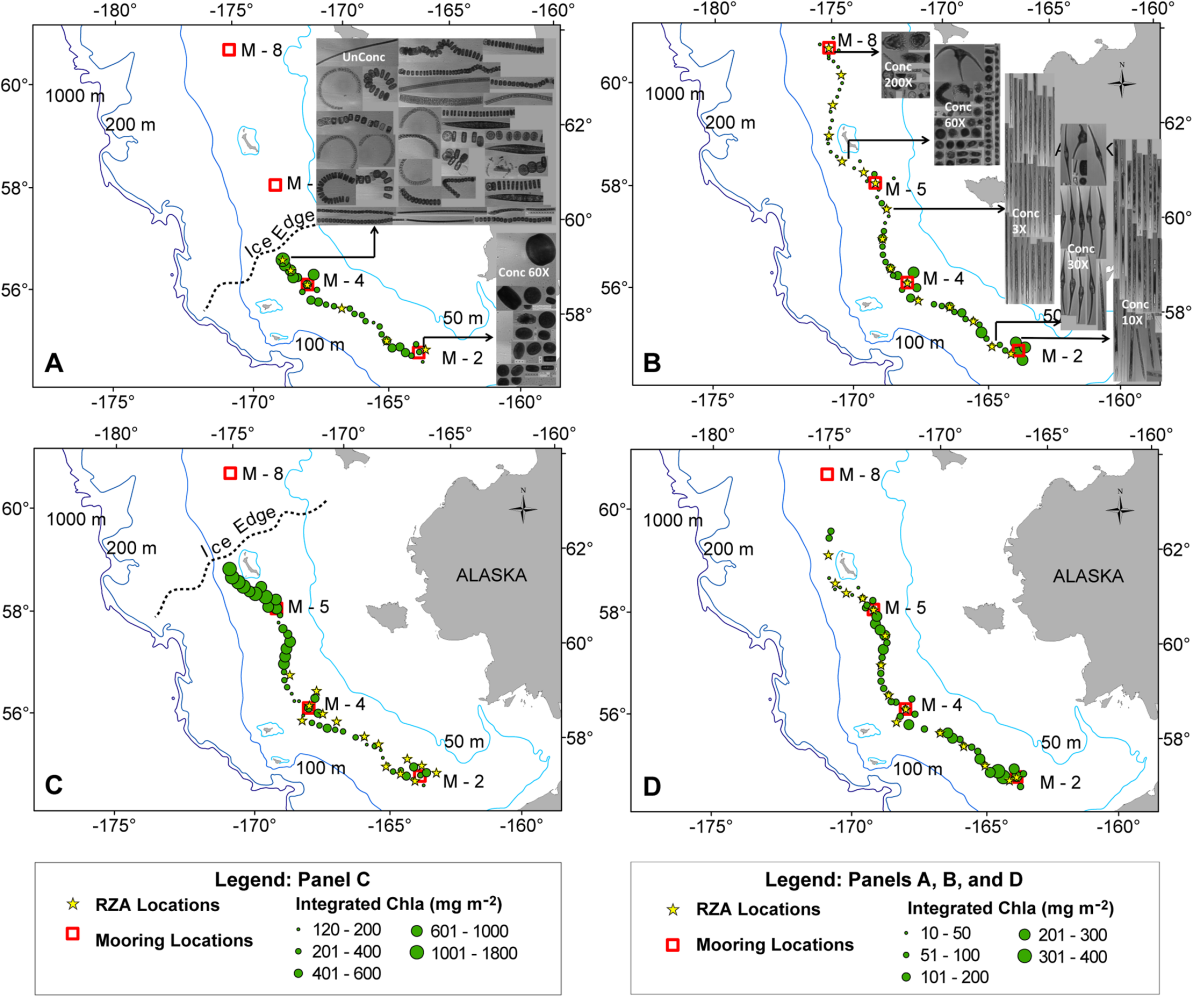
Phytoplankton. Integrated chlorophyll a (Chl*a*) in spring and autumn 2015 (A, B) and 2016 (C, D) collected along the 70 m isobath shows latitudinal and seasonal differences. Note difference in scale in spring 2016. Examples of large phytoplankton taxa (10 to 150 μm) in spring (A, inset) and autumn (B, inset) in 2015. Photos show cells in 3.5 ml of sample at the listed concentration. *Spring*, *south*: large phytoplankton were single cell centric diatoms (0.092 cells ml^-1^); *Spring*, *ice edge*: large phytoplankton were diatom chains of *Thalassiosira* sp., *Fragilariopsis* sp. and single pennate diatoms, *Pleurosigma* sp., (350 cells ml^-1^). *Summer*, *south*: large phytoplankton were centric diatoms (*Rhizosolenia* sp.) and dinoflagellates (*Ceratium* sp.); Summer, north: phytoplankton varied (small silicioflagellates (*Dictyocha* sp.), dinoflagellates (*Dinophysis* sp.), tintinnids, other taxa). Cell counts were 0.95, 0.29, 4.2, 0.32 and 0.060 cells ml^-1^ from south to north.

During spring in both years the integrated Chl*a* along the 70 m isobath increased substantially from the southeastern end of the isobath near Mooring 2 (M2) to the ice edge at the northeastern end of the sampled transect (Fig 2A and 2C). Sea ice presence prevented sampling north of ~57.5 ^o^N in spring 2015. Since ice extent was retracted in 2016, sampling that year continued further north until the ice edge was reached (~59 ^o^N). Highest concentrations of integrated Chl*a* were observed at stations closest to the sea ice edge in spring. From taxa-specific analyses (2015 only) it was noted that phytoplankton taxa consisted of sparse, small, single centric diatoms in open water at southern stations with biomass dominated by small cells (~40% of total Chl*a* was in the large-size fraction), while near the ice edge there were high numbers of long, centric diatom chains (largely *Thalassiosira*) including ice-associated taxa (e.g. *Fragilariopsis*) with the majority of the integrated biomass (~90%) in the large-size fraction. Chl*a* biomass in spring 2016 was substantially higher than in 2015, with bloom conditions (> 1 mg m^-3^ mean Chl*a*) observed along then entire 70-m isobath (Fig 2A and 2C). Integrated biomass was dominated by large phytoplankton (~ 89% of the total Chl*a* biomass at M2 to 92–96% near the ice edge, where Chl*a* reached maximum values >30 mg m^-3^). During late summer/ early autumn integrated Chl*a* was lower in the north than in the south (Fig 2B and 2D). Autumn-collected phytoplankton taxa were different than those observed in spring, and also varied latitudinally with relatively high Chl*a* and high numbers of large centric diatoms (*Rhizosolenia* sp.) and dinoflagellates (*Ceratium* sp.) in the south and low Chl*a* and low numbers of small silicoflagellates (*Dictyocha* sp.), dinoflagellaes (*Dinophysis* sp.) and other taxa in the north. *Pseudo-nitzchia* (smaller potentially toxic pennate diatoms potentially linked to warm water conditions) were observed at a few stations in the southern region. During late summer, in 2015 the large size fraction Chl*a* was ~85% of the biomass near M2 in the south and ~30% in the north. Thus, in both spring and late summer, higher Chl*a* was associated with a greater fraction of large (> 10 μm) phytoplankton, as observed in previous studies [23, 24].

### Zooplankton

**Fig 3:**
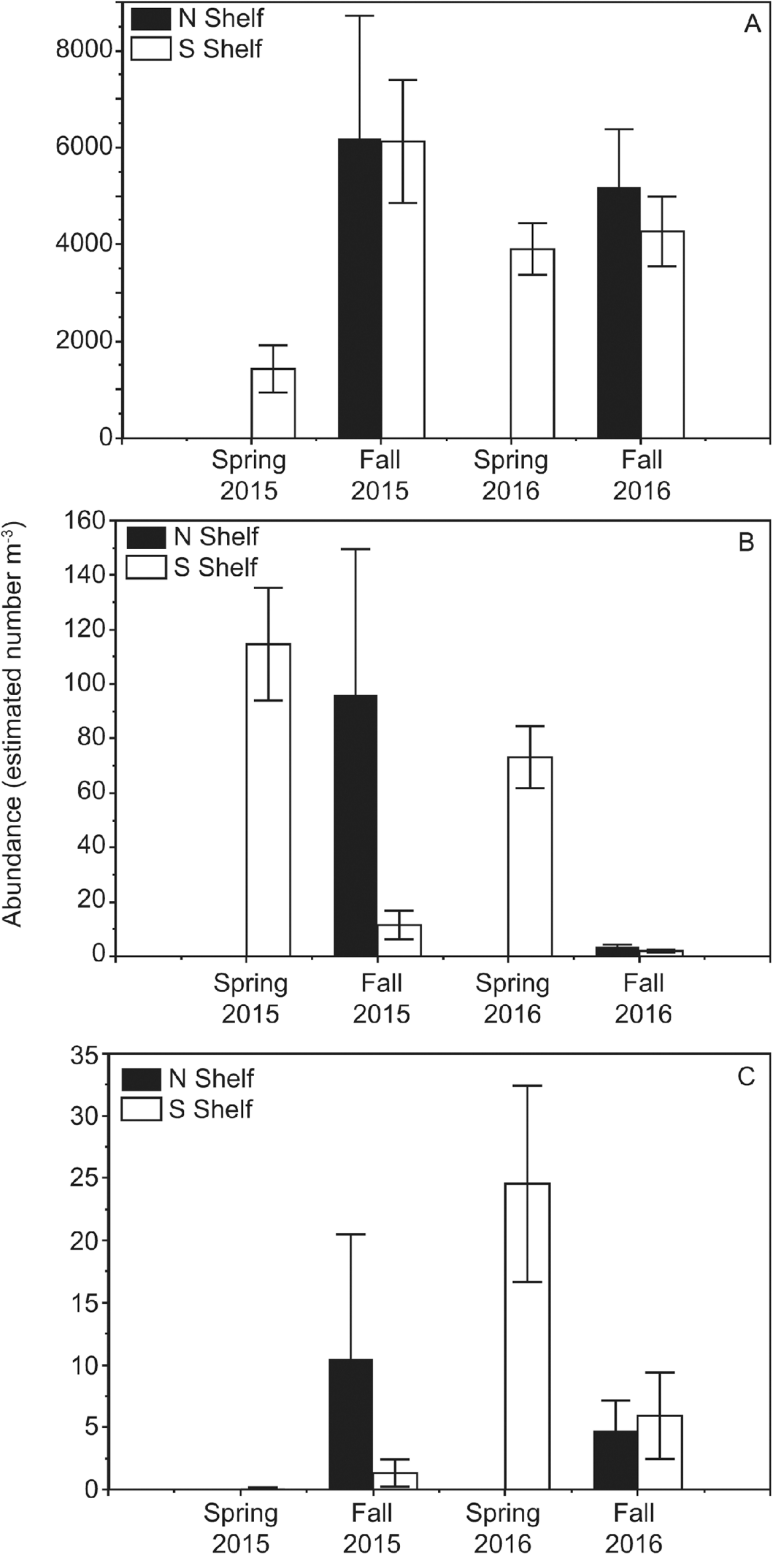
Zooplankton. Mean abundance (estimated number m^-3^) of small copepods < 2 mm (A), large copepods > 2 mm (B), and euphausiids < 5 mm (C) in the northern and southern shelf during spring and autumn of 2015–2016. Error bars represent ± standard error of the mean.

During spring of 2015, samples were collected only in the southern SEBS shelf as the presence of ice restricted sampling further north. During this time, the zooplankton community consisted of low abundances of small copepods (< 2mm) and high abundances of large copepods (> 2mm) (Fig 3A and 3B) in comparison to other years. Euphausiid (< 5mm) abundances were very low (Fig 3C). Autumn 2015 had similar abundances of small copepods across the entire shelf (Fig 3A), whereas large copepods and euphausiids were had higher abundances in the northern shelf (> 60°N) compared to the southern shelf (<60°N) (Fig 3B and 3C). The spring 2016 began with similar conditions with respect to large copepod abundance (Fig 3B), whereas small copepod and euphausiid abundances were higher than in 2015 (Fig 3A and 3C). The autumn of 2016 showed similar abundances of small copepods across the shelf, but very low abundances of large copepods (Fig 3A and 3B). Euphausiid abundances were lower in the autumn of 2016 compared to the spring, but were elevated across the southern shelf in particular compared to autumn 2015 (Fig 3C).

### Fish

**Fig 4:**
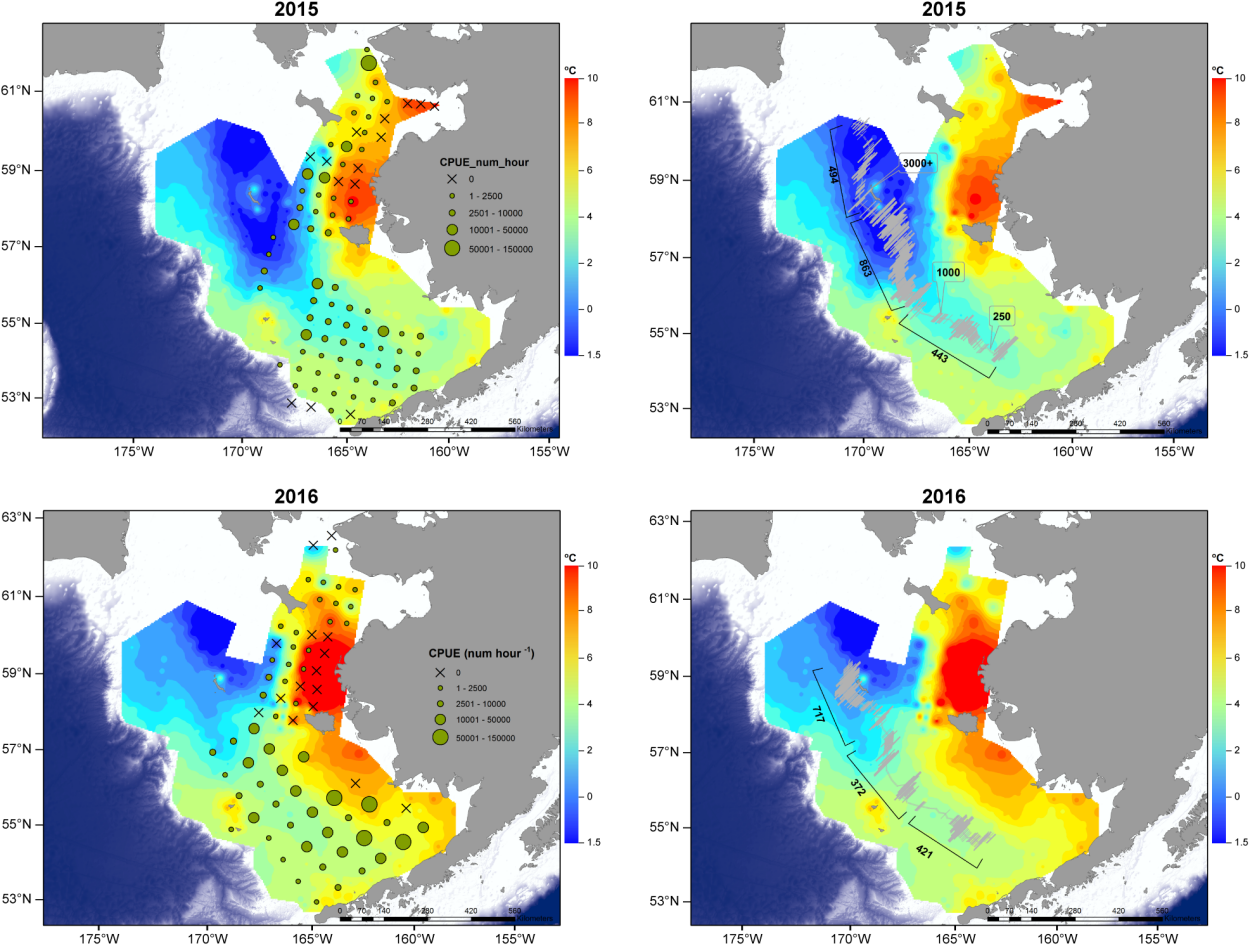
Fish catch. Catch (circles) of age-0 pollock (number h^-1^) as determined from oblique and surface trawling in 2015 (A) and 2016 (C). X indicates a trawl conducted but no catch. Heat map presents bottom temperatures (^o^C) over the southeast and Northern Bering Sea shelves. Cold Pool denoted where bottom temperatures <2 ^o^C (blue color ramp). Catches of age-0 pollock were low over the southern shelf in 2015, with higher catches that year in the vicinity of the Cold Pool (<2 ^o^C). In 2016 trawl catches of age-0 pollock over the southern shelf were higher than in 2015 and reduced in the north relative to the southern shelf. Acoustic backscatter (NASC, m^2^/nmi^2^) estimates in 2015 (B) and 2016 (D) indicate higher backscatter in the Cold Pool relative to the shelf in both years.

In 2015, gridded stations over the SEBS shelf had few age-0 pollock collected from oblique tows relative to the NBS (Fig 4A) but low numbers could also reflect changes in gear type and selectivity in 2015. Abundances of age-0 pollock from NBS in 2015 were well above average for that system (Table 2). However, severe weather limited trawl sampling over the full shelf extent in 2015, and no trawling was conducted over the northwest shelf. Notable differences in juvenile pollock distribution were observed in 2016 relative to 2015. In 2016, age-0 pollock were abundant in oblique trawls over the SEBS shelf but catches were lower from surface trawls conducted in the NBS (Fig 4C; Table 2), the opposite pattern as was observed the previous year. Comparison of surface trawl catches in 2014 (historical data) and 2016 indicated age-0 pollock biomass was consistent with catches observed during the previous Warm Year stanza (2001–2005), suggesting a similar pollock response to a Warm ecosystem.

**Table 2:** Walleye pollock abundance estimates.

Year	Southeast Shelf (Estimate)	Southeast Shelf (SD)	Northern Shelf (Estimate)	Northern Shelf (SD)
2002	18,420	7,017	4,175	1,659
2003	17,202	4,617	615	450
2004	81,956	19,896	6,400	1,830
2005	72,150	20,640	6,404	1,961
2006	9,735	1,937	109	73
2007	2,091	515	38	19
2008	3,763	1,339	–	–
2009	69	38	11	6
2010	571	180	15	9
2011	881	338	19	12
2012	766	157	3	3
2013	–	–	194	203
2014	22,801	5,175	34,126	20,045
2015	–	–	21,208	16,189
2016	17,135	4,885	1,548	1,593

Time series of annual mean age-0 pollock abundances (metric tonnes, +/- 1 standard deviation) from surface trawls conducted over the southeast Bering Sea and Northern Bering Sea shelves.–indicates no survey data from surface trawls.

Examination of acoustic data collected along a latitudinal gradient (70m isobath) revealed lower (2015: mean NASC of 443 m^2^/nmi^2^, 2016: mean NASC of 421 m^2^/nmi^2^) backscatter over the southeast shelf, with higher backscatter (2015: mean NASC of 863 m^2^/nmi^2^, 2016: mean NASC of 717 m^2^/nmi^2^) in the Cold Pool (Fig 4B and 4D). It is unlikely that these aggregations were jellyfish; acoustic backscatter sign was more consistent with that of pollock, (age-0, age-1 or mixed schools).

**Fig 5:**
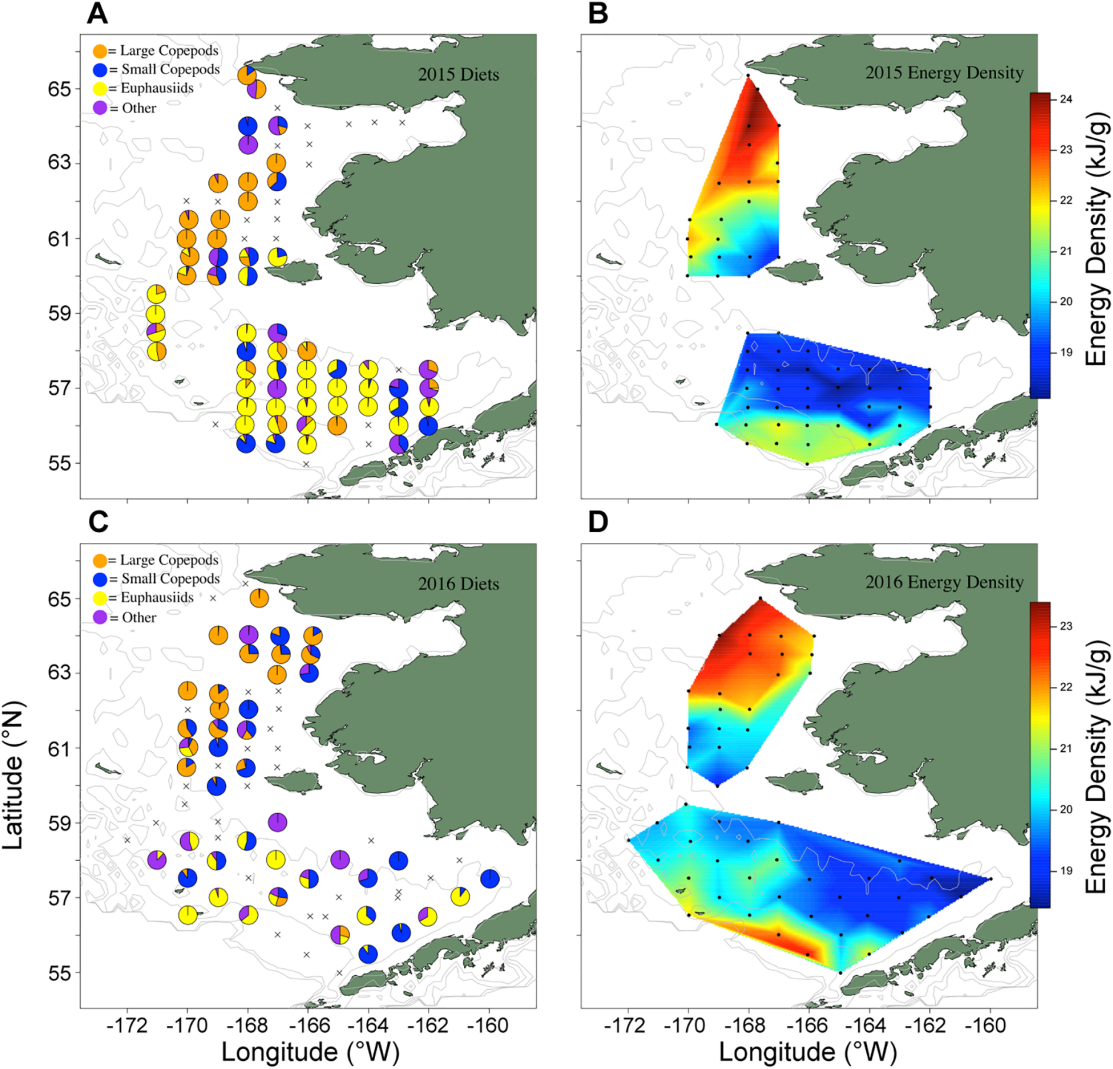
Diet and condition analyses. Stomach contents of age-0 pollock collected from trawl surveys over the southeast and Northern Bering Sea shelves (A: 2015, C: 2016). Diets over the southeastern shelf were comprised of euphausiids while large copepods made up the majority of the diets of fish collected in the Northern Bering Sea. Incidence of large copepods in the diet increased latitudinally, similar to observations field-collected zooplankton. Spatial heat map of age-0 pollock energy densities in 2015 (B) and 2016 (D). Heat map with color ramp denotes increasing energy densities. Energy densities were generally higher in the north relative to the south, possibly reflecting diet differences.

Diet analyses of age-0 pollock revealed spatial differences in feeding (Fig 5A and 5C). Over the southeastern shelf, stomach contents were primarily composed of euphausiids (*Thysanoessa* spp.; 55.6% in 2015, and 46.4% in 2016), though large and small copepods made up 9% and 17% of the total diet biomass, respectively in 2015 and 4.5% and 46.2% in 2016. Feeding of age-0 pollock collected from the NBS exhibited a strong latitudinal gradient in ingested prey type that was not observed in the south. At the southernmost edge (60.0–60.5 ^o^N), age-0 pollock consumed significant numbers of copepods, with small copepods (*Pseudocalanus* spp.) being more prevalent in the diets of fish collected in the warmer waters nearshore (east) and large copepods (*Calanus* spp.) more prevalent offshore (west) (Fig 5A and 5C). In the SEBS, euphausiids dominated but small and large copepods were present in meaningful proportions. In both years, pollock fed primarily on euphausiids on the SEBS shelf and on large copepods in the NBS.

**Fig 6:**
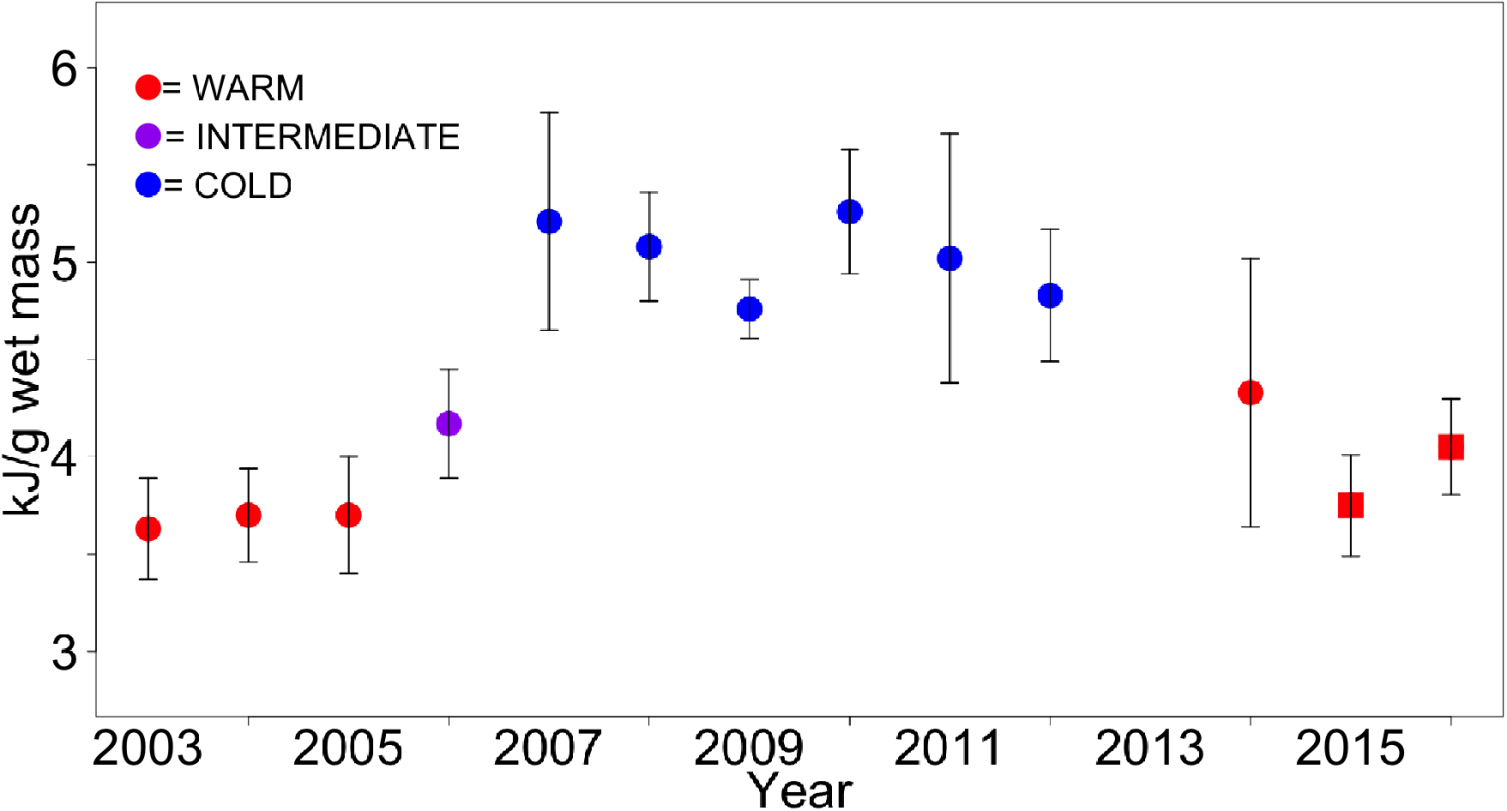
Energy content. Average energy densities of age-0 pollock collected from the southeast Bering Sea shelf during Warm (red), Average (purple) and Cold (blue years). Circles indicate data collected from surface trawls, squares indicate data collected from oblique trawls. Estimates from 2015 indicate a return to Warm Year bioenergetic status for age-0 pollock, though values were higher than during a previous Warm Year stanza. Estimates from 2016 were even higher than 2015, though not as high as during Cold Years. Results imply that age-0 pollock were able to forage on prey of sufficient quantity and quality to partially mitigate Warm Year effects on provisioning.

There was a latitudinal gradient in age-0 pollock energy content that was similar to patterns observed from diet analyses (Fig 5B and 5D), with highest energy densities noted in the northernmost reaches of the NBS, though some higher energy densities were noted in the southernmost SEBS as well (Fig 5B and 5D). Higher energy densities in the southernmost SEBS are likely age-0 pollock ingressed from the Gulf of Alaska through Unimak Pass. A time series of mean energy densities over the SEBS shelf indicated a decline in whole body energy content from the previous Cold Year phase (2007–2012) though energy densities were not as low as during the only other previous Warm Year stanza on record (2001–2005; Fig 6).

**Fig 7:**
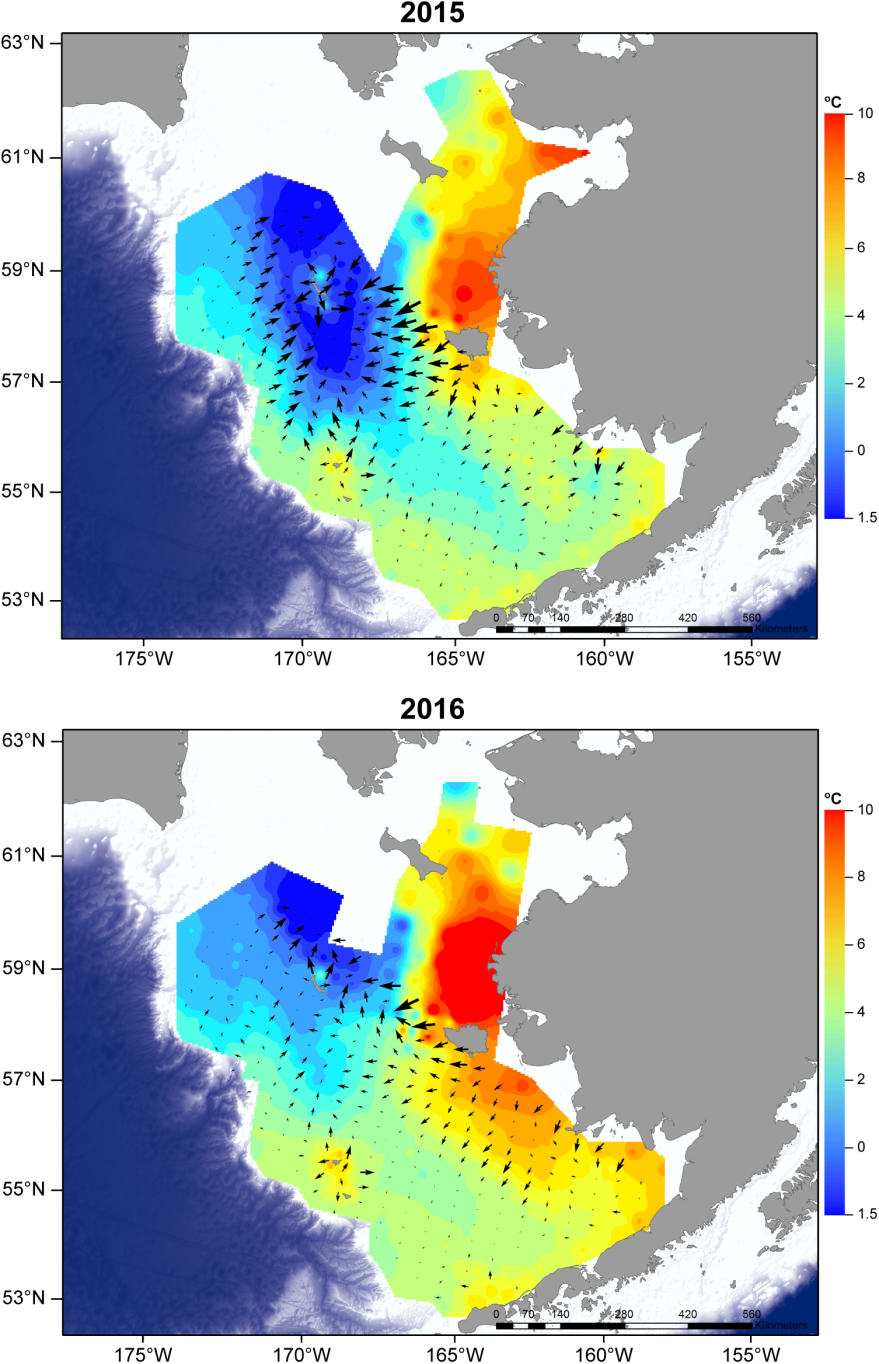
Thermal gradients. Vectors denoting strength of temperature gradients in 2015 (A) and 2016 (B). Note that vectors are adjusted to orient toward lower temperatures.

Analyses of thermal gradients over the Bering Sea shelf indicated thermal gradients were present in both years, but were stronger in 2015 compared to 2016 (Fig 7A and 7B). Particularly striking differences were noted over the middle domain of the SEBS shelf and in the vicinity of the Pribilof Islands and northward, where thermal gradients were strong in 2015 and noticeably weaker in 2016. In general, gradients were more strongly oriented toward the Cold Pool in 2015 forming a discernable central tendency in that year, while 2016 lacked this directed centralization around the Cold Pool.

## Discussion

We demonstrate that the ecosystems of the Northern Bering Sea and southeast Bering Sea shelves have responded to an extended Warm Year period (2014–2016). We noted that springtime levels of Chl*a* over the southern shelf were reduced relative to sea ice-covered areas to the north, and we observed a zooplankton community with fewer large copepods over the southeastern shelf, characteristic of Warm Years. Age-0 pollock abundances were higher in the north in 2015, higher in the south in 2016, and pollock had lower energy densities in both years than they did during a previous Cold Year stanza. However, we also demonstrate that an area of low-temperature, productive waters (Cold Pool) and/or the presence of sufficient larger sized prey may have provided a refuge from poor conditions over the southern shelf. We propose that the negative impact of the recent Warm Year stanza (2014–2016) on the Bering Sea pollock population may be mitigated by selective use of thermal refuges and alternative food sources. As such, juvenile pollock may be able to withstand an acute warming event and avoid the devastating effects on recruitment observed during the prior Warm Year stanza (2001–2005).

### Phytoplankton

Integrated Chl*a* differed substantially along a latitudinal gradient extending from the SEBS to the NBS. Chl*a* data from spring indicated higher phytoplankton biomass near the sea ice edge relative to southerly areas which remained ice free in winter. Phytoplankton community composition near the ice edge was characterized by long, centric diatom chains and ice-associated species. Ice-associated phytoplankton have been shown to be high in polyunsaturated fatty acids [25, 26] which are key to transfer of energy to upper trophic levels. In contrast, in 2015 the Chla biomass in open water was comprised of small phytoplankton (60% of biomass) and single-celled diatoms. We hypothesize that in spring, improved algal resources were available to the upper trophic communities occurring near the Cold Pool while communities outside of the Cold Pool experienced less algal production. The higher phytoplankton biomass (both total and in the large size fraction) observed near the ice and in open water in spring 2016 may have enhanced the overall ecosystem productivity compared to 2015. In autumn however, we noted that Chl*a* concentrations were higher at the southernmost extent of the sampling and reduced at northern latitudes, a trend opposite to that observed during spring. High Chl*a* concentrations over the southern shelf are related to wind mixing, nutrient input, and below-pycnocline temperatures [23], but could also reflect the cumulative effects of differential grazing between the southern and northern most extents of the sampling.

### Zooplankton

Small copepods (*Acartia* spp., *Oithona* spp., and *Pseudocalanus* spp.) were numerically dominant over the SEBS shelf as has been observed by others [4, 27, 28, 29]. Numbers of small copepods remained similar across the SEBS shelf during 2015–2016, with the exception of the colder spring of 2015. Large copepods (*Calanus marshallae/glacialis*) were numerically abundant during the spring of 2015 on the southern shelf where sea ice was present. In contrast, euphausiid (*Thysanoessa* spp.; typically 3–15% lipid [5]) abundance was low in 2015. During the autumn of 2015, large copepod abundance declined on the southern SEBS shelf, but remained high in the northern portion of the SEBS shelf. This pattern was mirrored by the euphausiid abundance and coincided with the retraction of the Cold Pool northward in the summer of 2015. Therefore, by autumn 2015, feeding conditions for young fish over the southern shelf was reduced relative to conditions to the north. In 2016, spring sea ice extent was retracted and RZA sampling over the SEBS shelf did not approach the ice edge. Both large zooplankton and euphausiids remained present during the spring of 2016. In the autumn, large copepods were absent from the shelf, consistent with other observations during warm periods [4, 29, 30]. Euphausiid abundance also declined, but remained detectable across the shelf providing a potential food source for age-0 pollock occurring there. The lack of larger-sized zooplankton (*Calanus marshallae/glacialis* and *Thysanoessa* spp.) during autumn is critical because age-0 pollock are provisioning during this period to ensure overwinter survival [30].

### Fish

Abundances of juvenile pollock over the SEBS shelf were low in 2015 but appeared to be higher in the north and associated with the Cold Pool, as evidenced by higher trawl catches at the Cold Pool periphery and acoustic data collected from within the Cold Pool. In 2016, abundances of pollock in trawl catches were higher over the SEBS shelf than in 2015, but acoustic backscatter data also indicated fish presence in the Cold Pool. We hypothesize that juvenile pollock have the potential to buffer the adverse influence of sequential Warm Years either by utilizing the Cold Pool and its associated productivity when possible, or when an energy-rich food source, euphausiids, are available. Utilization of the Cold Pool provides a dual benefit: reduction in metabolic expenditure, and intake of higher quality prey allowing young fish to adequately provision for overwinter [31].

### Utilization of the Cold Pool

Unlike adults [32, 33], juvenile pollock have flexible thermal ranges that allow them to tolerate low temperatures [34, 35]. Accordingly, young pollock have the potential to tolerate the frigid, ice-influenced waters of the Cold Pool and exploit the more productive waters associated with it. There are at least two mechanisms by which juvenile pollock could realize benefits derived from Cold Pool associations during Warm Year stanzas. In the first, differential mortality experienced by juvenile pollock subpopulations could grant disproportionate survivorship to those individuals that occur in the vicinity of the Cold Pool, while selectively removing those individuals that are constrained to the SEBS shelf. Differential mortality would be a function of temperature and prey availability. It is well-known that there are at least three distinct pollock spawning aggregations in the SEBS, an aggregation at Bogoslof Island, and aggregation along the Alaska Peninsula in the vicinity of Unimak Island, and an aggregation around the Pribilof Islands [36, 37]. Work to model larval pollock drift trajectories from these three spawning locations suggests variable transport pathways and differential connectivity with juvenile nursery areas over the continental shelf [38]. Indeed, modeling work has demonstrated that the NBS, in particular the middle and outer shelves 58–60 ^o^N (St. Matthew Island and environs), is a significant nursery sink to larvae derived from the Pribilof Islands spawning population, particularly during Warm Years. In contrast, connectivity to the SEBS shelf is realized primarily from dispersal of larvae originating from the Alaska Peninsula spawning aggregation [38]. If larvae, and subsequently age-0 juveniles, remain constrained to their respective areas of delivery, we would expect that during Cold Years when the Cold Pool penetrates the SEBS shelf, both subpopulations experience the lower metabolic rates that are associated with Cold Pool temperatures and both would have access to prevalent Cold Year large crustacean zooplankton (high quality prey). In Warm Years however, when the Cold Pool extent is limited to the north, only the northerly subpopulations of pollock would benefit from its increased productivity; populations over the SEBS shelf would be consigned to warm waters and a prey field lacking in large-sized copepods. As such, we might expect differential autumn provisioning and overwinter survivorship between the subregions with higher survival attributed to northerly populations during Warm Year stanzas.

The second mechanism by which pollock could exploit resources in the Cold Pool is via movement from one region to another, and juvenile pollock are capable of swimming large distances. Laboratory work demonstrates that routine swimming speeds of small-sized age-0 pollock juveniles (10–20 mm total length) are ~80 mm s^-1^ at 2 ^o^C [39]. At that rate, a small pollock could travel ~200 km in a month, while larger-sized juveniles (~60–80 mm TL,) such as those collected during our surveys, might travel even further, especially if they take advantage of the directional baroclinic flow (~5–8 cm s^-1^) that runs northward along the 100 and 200 m isobaths (middle and outer domains)[40, 41]. The rough estimate of travel potential, coupled with the observation that juvenile pollock spatial distributions are not limited by temperature [42, 43], make it realistic to postulate directed movement of juvenile pollock from the SEBS shelf to the Cold Pool when resources over the southern shelf are limiting.

Of course, thermal cues must be present at sufficient levels to prompt pollock movement toward the Cold Pool. While this study cannot conclusively identify thermal response thresholds, our analyses of temperature gradients indicated that gradients were present in both years. Interestingly, gradients were stronger in 2015 over the SEBS shelf, which could indicate more robust cues for migration following temperature clines. We note that pollock biomass in trawls over the SEBS shelf was lower in 2015 compared to 2016 but elevated in the NBS, a trend consistent with a pattern of movement from the warm, less productive southern shelf to the more productive Cold Pool. Gradients over the southern shelf were markedly weaker in 2016 and pollock biomass in SEBS trawls were high while trawl catches in the NBS were reduced. This could indicate reduced movement from the southern shelf in 2016 due to weaker temperature gradient cues. Still, backscatter estimates indicated elevated scattering estimates in the Cold Pool in both years, suggesting at least some utilization of the Cold Pool in 2016 as well as 2015.

### Utilization of alternative prey

Estimates of pollock energy densities indicated improved fish condition in the NBS relative to the SEBS shelf in 2015; however, pollock energy densities were more similar between the two regions in 2016. Improved pollock condition in the NBS in 2015 can be explained by better foraging in north relative to the warm SBES shelf in that year, i.e. high phytoplankton production and the presence of large copepods associated with the Cold Pool. Pollock did not appear to associate with the Cold Pool as strongly in 2016, remaining on the southern portion of the shelf. Large copepod abundances were extremely low over the southern shelf in 2016; however, euphausiid abundances were higher in this region relative to 2015 perhaps related to the high biomass of large phytoplankton in spring (early May), offering a high quality prey item important for pollock growth and provisioning. Euphausiids are preferred prey for larger sized juvenile pollock and diet data indicated fish utilized this food source. Thus, the higher energy densities observed in 2016 should have been surprising in the absence of large copepod prey availability but they are consistent with the notion of use of euphausiid prey to buffer the effects of Warm Year conditions.

### Pollock recruitment

Recruitment of the 2015 year class to age-1 (assessed in 2016) was higher than expected for a second consecutive Warm Year. Stock assessment estimates indicate recruitment from the 2015 year class was level with estimates of recruitment of the 2014 year class. As reference, between 2002 and 2003, the second and third years of the previous Warm Year stanza, recruitment of age-0 pollock to age-1 had declined 40% [1]. Level recruitment is consistent with the notion that age-0 pollock were able to buffer some of the Warm Year effects, either by refuging in the Cold Pool (2015) or by consuming euphausiids over the SEBS shelf (2015 and 2016). Survival estimates of the overwinter success of the 2016 cohort are not yet available, but data from our study indicate it could again be level, which is surprising during a third consecutive Warm Year. We anticipate moderated effects on recruitment, blunting the devastating population declines observed during the last Warm Year period. Based on historical data, we postulate that the dramatic declines 2001–2005 manifested due to a retracted, inaccessible Cold Pool [44], and fewer euphausiids available over the SEBS shelf and in the diets of age-0 pollock [45, 46].

## Conclusions

The Bering Sea experienced sustained ocean warming 2014–2016, and we present evidence that the ecosystem has responded to the new stanza of Warm Years. Poor ecosystem conditions during Warm Years have been followed by reduced recruitment of age-0 pollock and the risk of significant decline in the pollock population in 2–3 years. However, we noted juvenile pollock were associated with high quality prey (large copepods) and lower temperatures in the Cold Pool during 2015 and they consumed euphausiid prey over the southern shelf in both 2015 and 2016. Both observations indicate that pollock may have been able to buffer against poor conditions and ameliorate the anticipated poor recruitment. Presently, there is evidence of cooler conditions over the SEBS shelf as sea ice has formed primarily along the coastline of the Alaska mainland, descending as far as 58 ^o^N (March 2017). Ocean temperatures derived from year-round moorings indicate bottom temperatures ~3 ^o^C. We suggest that, if pollock were successfully able to mitigate Warm Year effects in 2015 and 2016, and if conditions remain cooler in 2017, the Bering Sea pollock population may not experience the dramatic declines noted during the previous Warm Year stanza.

## Supporting information

S1 Table Temperature. (XLS)

S2 Table Chlorophyll. (XLSX)

S3 Table Zooplankton. (XLS)

S4 Table Pollock. (XLS)

S5 Table Backscatter. (XLSX)

S6 Table Diets. (XLS)

S7 Table Energy. (XLS)
